# Timing of dental surgery in patients receiving bone-modifying agents: Medication related osteonecrosis of jaw (MRONJ) and implant outcomes in a cohort of 5,284 oncology patients within an integrated dental pathway

**DOI:** 10.4317/jced.63634

**Published:** 2026-01-28

**Authors:** Asif Ahmed, P.V. Jain, Mutum Sangeeta Devi

**Affiliations:** 1 BDS,MDS; Associate Consultant (Prosthodontist); Department of Dental Oncology &amp; Maxillofacial Prosthetics, Tata Medical Center, Kolkata, India; 2 MBBS, MS, DNB; Associate Consultant; Department of Head &amp; Neck Surgery, Tata Medical Center, Kolkata, India; 3 BDS, MDS; Dental Oncologist; Department of Dental Oncology &amp; Maxillofacial Prosthetics, Tata Medical Center, Kolkata, India

## Abstract

**Background:**

Medication-related osteonecrosis of the jaws (MRONJ) is an important toxicity of bone-modifying agents (BMAs). Guidelines recommend dental assessment and risk reduction before high-dose antiresorptive therapy, but real-world data on outcomes by timing of dental procedures relative to BMA therapy remain limited [1-4]. This study evaluated MRONJ and implant outcomes according to the timing of dentoalveolar surgery within an integrated oncology-dental pathway.

**Material and Methods:**

We retrospectively reviewed 5,284 consecutive patients referred for dental evaluation in the context of planned or ongoing BMA therapy from January 2019 to December 2023 at a tertiary centre. Primary diagnoses were breast cancer (n=3,611), multiple myeloma (n=1,305), lung cancer (n=300) and prostate cancer (n=68). BMAs included intravenous zoledronic acid (n=4,087), subcutaneous denosumab (n=1,187) and oral alendronate (n=10). Patients were categorised as pre-BMA (all invasive dental procedures completed before first BMA dose), intra-BMA (procedures during therapy within a coordinated 6-month "drug-holiday" window) or post-BMA (6 months after BMA completion). Outcomes were MRONJ (AAOMS criteria) and patient-level implant failure (1 failed implant per patient) with 18 months of follow-up. Chi-square tests and multivariable logistic regression were applied.

**Results:**

Overall, 4,025 patients (76.2%) were managed pre-BMA, 1,001 (18.9%) intra-BMA and 258 (4.9%) post-BMA. Extractions were performed in 1,448 patients (27.4%): 968 pre-BMA, 445 intra-BMA and 35 post-BMA. No MRONJ occurred after pre-BMA extractions (0/968), compared with 6/445 intra-BMA extraction patients (1.35%) and 2/35 post-BMA extraction patients (5.71%; ²=27.3, p1.2×10-6). Overall MRONJ incidence was 12/5,284 (0.23%), including 8 post-extraction cases, 1 case after implant placement intra-BMA and 3 spontaneous cases without preceding dentoalveolar surgery. A total of 366 patients received 915 implants. At the patient level, implant failure occurred in 45/366 implant patients (12.3%): 16/270 pre-BMA (5.9%), 20/71 intra-BMA (28.2%) and 9/25 post-BMA (36.0%; ²=39.8, p2.3×10-9). In multivariable models, pre-BMA implant placement was independently associated with substantially lower odds of any implant failure than intra-BMA placement (adjusted odds ratio [OR] 0.16; 95% confidence interval [CI] 0.08-0.33; p&lt;0.0001), whereas post-BMA implants had similar odds of failure to intra-BMA implants (OR 1.43; 95% CI 0.55-3.77; p=0.46). Pre-BMA timing was also associated with lower odds of undergoing extractions than intra- or post-BMA management.

**Conclusions:**

Within an integrated oncology-dental pathway, completing necessary extractions and implants before BMA initiation, with adequate healing, was associated with no postsurgical MRONJ and the lowest patient-level implant failure rates. Dental surgery during or after BMA therapy, even with planned drug holidays, was associated with higher MRONJ and implant loss. Early dental referral and a cautious approach to elective implants after BMA initiation should be standard in oncology supportive care.

## Introduction

Bone-modifying agents such as zoledronic acid and denosumab are widely used to prevent skeletal-related events (SREs) in patients with metastatic breast cancer, multiple myeloma and other solid tumours (1 - 4). They reduce pathologic fractures, spinal cord compression and the need for radiotherapy or surgery to bone, and are therefore central to supportive oncology care. Soon after the introduction of high-potency intravenous bisphosphonates, clusters of jaw necrosis were reported, initially termed bisphosphonate-related osteonecrosis of the jaw and now encompassed under medication-related osteonecrosis of the jaws (MRONJ) (5 - 7). MRONJ is linked to antiresorptive and antiangiogenic agents and, although uncommon, can be painful, function-limiting and difficult to treat (8 - 11). Major position papers and clinical practice guidelines emphasise pre-treatment dental assessment, elimination of oral foci of infection, avoidance of elective dentoalveolar surgery during high-dose BMA therapy and cautious consideration of dental implants (3 , 4 , 8 , 12). Real-world data indicate that MRONJ risk is influenced by cumulative BMA exposure, type of malignancy, concomitant treatments and local dental practice (11 , 13 - 16). Dentoalveolar surgery, particularly tooth extraction, is the most frequent precipitating factor (12 , 17 , 18). However, clinicians still face practical questions: How safe is it to perform extractions or implants before BMA therapy with a defined healing interval? What are the risks when procedures are needed during therapy, even with drug holidays? Is it reasonable to place implants after BMA completion? We implemented an integrated oncology-dental pathway for patients considered for or receiving BMAs. The primary objective of this study was to describe MRONJ incidence and patient-level implant failure by timing of dental surgery relative to BMA therapy (pre-, intra-, post-BMA). A secondary objective was to identify independent predictors of extractions, implant placement and implant failure using multivariable logistic regression.

## Material and Methods

**Figure 1:**
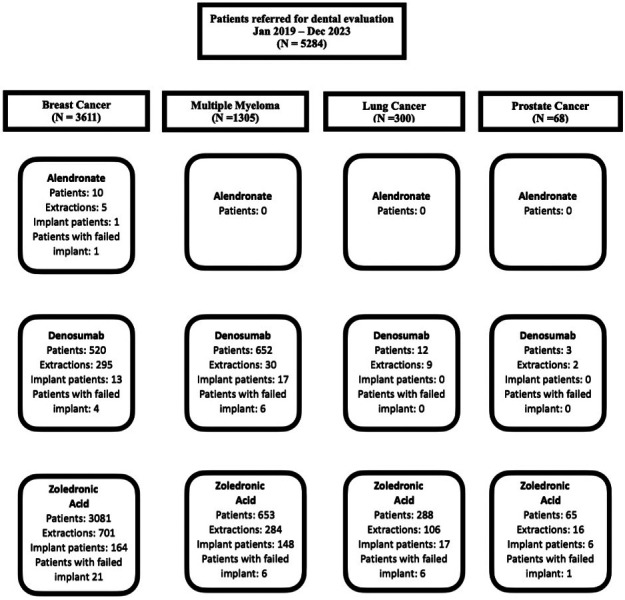
Study Consort Diagram.

- Study design and population This was a retrospective cohort study of all consecutive patients referred for dental evaluation in the context of planned or ongoing BMA therapy between January 2019 and December 2023 at a single tertiary cancer centre. Eligible patients had a diagnosis of breast cancer, multiple myeloma, lung cancer or prostate cancer; underwent baseline dental assessment as part of an institutional MRONJ-prevention protocol; and had at least 18 months of follow-up after their last invasive dental procedure. Patients with incomplete dental or follow-up records were excluded, (Fig. 1).

1

The study was approved by the Institutional Ethics Committee (insert reference number). The requirement for individual written informed consent was waived according to local regulations. - BMA exposure and timing groups BMAs included: Intravenous zoledronic acid (4 mg), Subcutaneous denosumab (120 mg), Oral alendronate (70 mg weekly). These agents were used according to contemporary indications for metastatic bone disease and multiple myeloma (1 - 4). Patients were allocated to one of three groups based on the timing of invasive dental procedures (tooth extractions and implant placement) relative to BMA therapy: 1. Pre-BMA: All indicated invasive dental procedures completed before the first BMA dose. BMA therapy commenced after mucosal healing, generally 6 weeks after the last extraction. In 27 patients with delayed healing, therapy was deferred to approximately 8 weeks. 2. Intra-BMA: Invasive dental procedures performed during ongoing BMA therapy, scheduled within a coordinated 6-month "drug-holiday" window (approximately 3 months before and 3 months after an expected BMA dose) agreed with the treating oncologist. 3. Post-BMA: Invasive dental procedures performed 6 months after completion of BMA therapy (range, 6-24 months). - Dental assessment and extractions All patients underwent thorough clinical examination and panoramic radiography at baseline. Teeth were indicated for extraction when they had advanced caries, severe periodontal disease, non-restorable endodontic lesions, root fractures or symptomatic impacted teeth. Extractions were carried out under local anaesthesia using atraumatic techniques, smoothing of sharp bony margins and primary closure when feasible. A standard regimen of ciprofloxacin 500 mg orally twice daily plus vitamin E 400 mg once daily was prescribed for 2 weeks before and 2 weeks after surgery, in line with previous protocols aimed at reducing infectious and oxidative stress components of MRONJ (11 , 17). Patients were reviewed at 1 week and 1 month post-extraction, then at least every 3 months for a minimum of 18 months. - Implant placement and prosthetic rehabilitation Dental implants were considered for patients with: controlled systemic disease, completed cancer staging and treatment plan, good oral hygiene and motivation, no previous MRONJ. Titanium screw-type implants were placed under local anaesthesia using standard drilling protocols. The same antibiotic and vitamin regimen was applied as for extractions. In intra-BMA patients, implants were scheduled within the drug-holiday window to avoid BMA administration close to the surgical procedure. Implants were prosthetically loaded after clinical stability and radiographic evidence of osseointegration. All successful implants received fixed or removable prostheses and were followed for at least 12 months after loading. - Definitions and outcomes MRONJ was defined according to the AAOMS 2022 criteria: exposed bone or bone that can be probed through an intraoral or extraoral fistula in the maxillofacial region persisting for more than 8 weeks, current or previous treatment with a BMA, and no history of radiation therapy to the jaws or obvious metastatic disease to the jaws (3). MRONJ cases were classified as: Post-extraction, Post-implant, Spontaneous (no dentoalveolar surgery in the preceding months). Implant failure was analysed at the patient level. A patient was classified as having implant failure if any implant: became mobile, required removal due to pain, infection or lack of osseointegration, or showed progressive radiographic bone loss leading to loss of function. Patients with multiple failed implants were counted once. - Statistical analysis Categorical variables were summarised as numbers and percentages. Chi-square tests were used to compare: extraction rates, implant placement rates, patient-level implant failure rates, between timing groups and across cancer types. One multivariable logistic regression models were fitted: Model 1- outcome = at least one failed implant (yes/no) among implant recipients. Predictors were: Timing group (pre-, intra-, post-BMA), Primary cancer site (breast, myeloma, lung, prostate), BMA agent (zoledronic acid, denosumab, alendronate). Indicator coding was used, with intra-BMA, breast cancer and alendronate as reference categories. Given the very small alendronate subgroup (n=10), estimates for BMA type were interpreted with caution. A simplified model including timing only was also fitted for implant failure to improve stability. Results are reported as odds ratios (ORs) with 95% confidence intervals (CIs) and p-values from Wald tests. Two-sided p&lt;0.05 was considered statistically significant. Statistical analyses were performed using Stata.

## Results

Patient characteristics and BMA exposure Among 5,284 patients, primary diagnoses were: Breast cancer: 3,611 (68.4%), Multiple myeloma: 1,305 (24.7%), Lung cancer: 300 (5.7%), Prostate cancer: 68 (1.3%). BMAs used were: Zoledronic acid: 4,087 (77.3%), Denosumab: 1,187 (22.5%), Alendronate: 10 (0.2%). Most myeloma patients received monthly BMA administration, whereas patients with solid tumours typically received 6-monthly dosing, consistent with current practice (1 - 4). Timing groups were: Pre-BMA: 4,025 (76.2%), Intra-BMA: 1,001 (18.9%), Post-BMA: 258 (4.9%). Table 1 can summarise diagnoses, BMA types and timing groups.

Table 1

Extractions and MRONJ Overall, 1,448/5,284 patients (27.4%) underwent tooth extractions: Pre-BMA: 968/4,025 (24.1%), Intra-BMA: 445/1,001 (44.5%), Post-BMA: 35/258 (13.6%). Extraction rates differed significantly between timing groups (p&lt;0.0001) and varied by cancer site (highest in myeloma, lowest in prostate; Table 2).

Table 2

**Figure 2:**
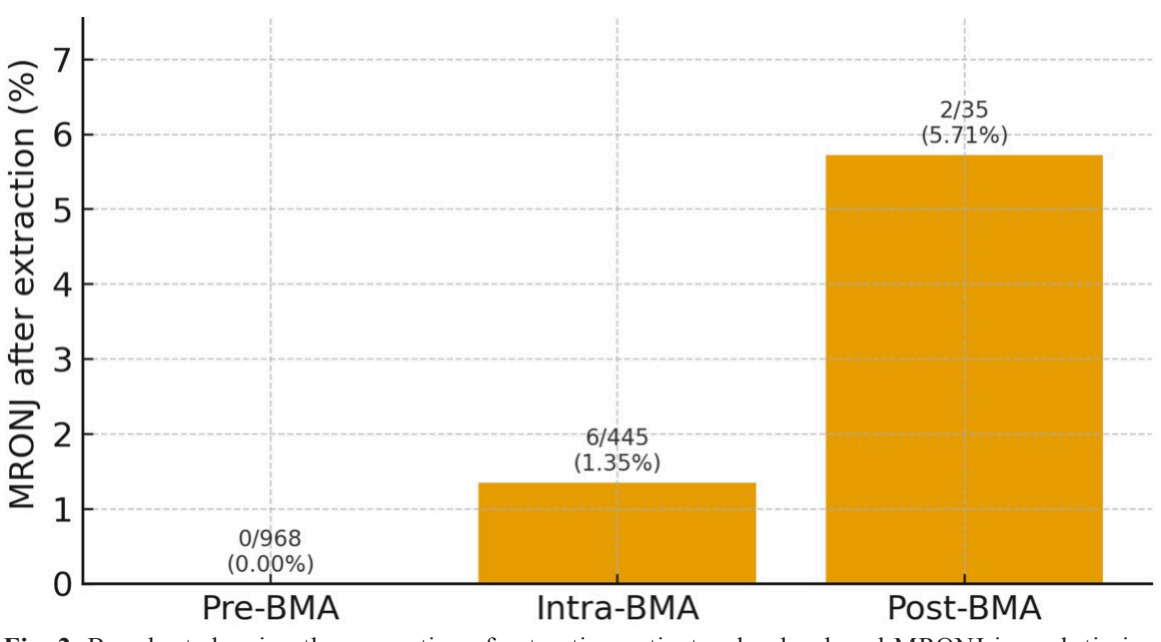
Bar chart showing the proportion of extraction patients who developed MRONJ in each timing group: pre-BMA (0/968; 0.0%), intra-BMA (6/445; 1.35%) and post-BMA (2/35; 5.71%). Error bars represent 95% confidence intervals. A significant difference in MRONJ incidence among groups was observed (χ2 =27.3, p ≈1.2x10-6), driven by higher risk whe extractions were performed during or after BMA therapy.

Eight MRONJ cases were temporally associated with extractions: Pre-BMA: 0/968 (0.0%), Intra-BMA: 6/445 (1.35%), Post-BMA: 2/35 (5.71%). MRONJ incidence after extraction differed significantly by timing (²=27.3, degrees of freedom [df]=2, p1.2×10-6), (Fig. 2).

2

**Figure 3:**
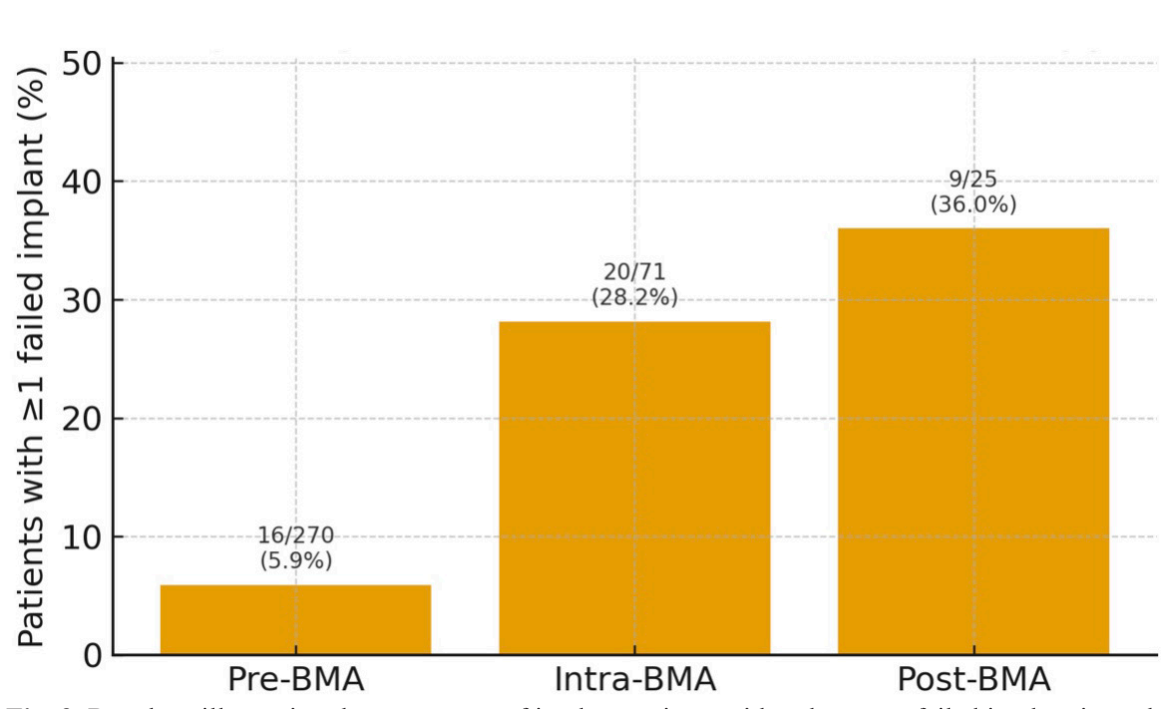
Bar chart illustrating the percentage of implant patients with at least one failed implant in each timing group: pre-BMA (16/270; 5.9%), intra-BMA (20/71; 28.2%) and post-BMA (9/25; 36.0%). Error bars represent 95% confidence intervals.

In total, 12/5,284 patients (0.23%) developed MRONJ: 8 cases following extraction, 1 case after implant placement during intra-BMA, 3 spontaneous cases in patients on, or with a history of, BMA therapy without recent dentoalveolar surgery. Implant placement and patient-level failure A total of 366 patients received 915 implants: (Fig. 3).

3

Pre-BMA: 270/4,025 patients (6.7%), Intra-BMA: 71/1,001 (7.1%), Post-BMA: 25/258 (9.7%). Implant placement rates varied with cancer type, being proportionally more frequent in myeloma and prostate candidates (Table 3).

Table 3

At patient level, implant failure occurred in 45/366 implant patients (12.3%): Pre-BMA: 16/270 (5.9%), Intra-BMA: 20/71 (28.2%), Post-BMA: 9/25 (36.0%). Differences in failure rate across timing groups were highly significant (²=39.8, df=2, p2.3×10-9; Table 4).

Table 4

Multivariable analysis Predictors of patient-level implant failure (implant recipients) In the simplified model including 366 implant patients and timing only (Table 5), using intra-BMA as reference: Pre-BMA implant placement was strongly protective (OR 0.16; 95% CI 0.08-0.33; p&lt;0.0001). Post-BMA implant placement had similar odds of failure to intra-BMA (OR 1.43; 95% CI 0.55-3.77; p=0.46).

Table 5

## Discussion

In this large real-world cohort of 5,284 oncology patients exposed or planned to be exposed to BMAs, overall MRONJ incidence was low (0.23%), but the timing of dental surgery relative to BMA therapy had a major impact on outcomes. The most striking observation was that no MRONJ occurred after pre-BMA extractions despite 968 extraction patients and at least 18 months' follow-up. In contrast, MRONJ occurred in 1.35% of intra-BMA extraction patients and 5.71% of post-BMA extraction patients. These data reinforce the concept that the combination of cumulative antiresorptive exposure and dentoalveolar surgery is a key risk factor for MRONJ (8 , 12 , 17 - 20). Our findings are concordant with prospective and population-based studies linking MRONJ incidence to BMA exposure patterns and extractions (11 , 13 , 14 , 18). At the same time, the gradient in patient-level implant failure was clinically important: 5.9% in pre-BMA implant patients versus 28.2% intra-BMA and 36.0% post-BMA. The multivariable model confirmed that pre-BMA implant placement was independently associated with markedly reduced odds of failure compared to intra-BMA placement, whereas post-BMA implants behaved similarly to implants placed during therapy. Evidence regarding implants in cancer patients treated with high-dose BMAs is limited and heterogeneous, and guidelines currently urge caution or discourage implants during active high-dose therapy (3 , 4 , 8 , 21). Our results support limiting implants to carefully selected patients and strongly favouring pre-BMA placement with adequate healing time. The integrated oncology-dental pathway implemented here-automatic referral before BMA, standardised extraction protocols, antibiotic and antioxidant prophylaxis, and coordinated "drug-holiday" scheduling-likely contributed to the low overall MRONJ incidence, similar to other series where proactive dental care reduced MRONJ rates (11 , 13 , 22). This model may be especially relevant in healthcare systems with high BMA use and limited access to specialised oral surgery services. Limitations This study has limitations. It is retrospective and single-centre, which may limit external generalisability. We did not capture all known risk modifiers, such as smoking, diabetes, corticosteroid dose or detailed oral hygiene indices, and residual confounding cannot be excluded (8 , 11 , 19 , 20). The alendronate group was very small, restricting comparisons between specific BMAs. The absolute number of MRONJ events was low, precluding robust multivariable analyses specifically for MRONJ, and survival or time-to-event analyses were not feasible with the available data. Nonetheless, the large sample, consistent gradients and alignment with existing mechanistic and epidemiological evidence (5 - 8 , 12 - 18 , 21) support the robustness of the main conclusions.

## Conclusions

In this cohort of patients receiving or planned for BMA therapy, completing extractions and implants before BMA initiation, with adequate healing, was associated with no postsurgical MRONJ and the lowest patient-level implant failure rate. Dental surgery during or after BMA therapy-even with coordinated drug holidays-was associated with higher MRONJ incidence and substantially increased implant failure. These findings strongly support early dental referral for patients in whom BMAs are being considered, systematic elimination of oral foci before high-dose antiresorptive therapy and a conservative, individualised approach to implants once BMA exposure has begun or completed.

## Figures and Tables

**Table 1:** Baseline oncologic diagnosis, bone-modifying agent exposure and timing of dental management (N = 5,284).

Variable	n	% of total
Primary diagnosis		
Breast cancer	3,611	68.4
Multiple myeloma	1,305	24.7
Lung cancer	300	5.7
Prostate cancer	68	1.3
Primary bone-modifying agent		
Zoledronic acid (IV)	4,087	77.3
Denosumab (SC)	1,187	22.5
Alendronate (oral)	10	0.2
Timing of dental management		
Pre-BMA	4,025	76.2
Intra-BMA	1,001	18.9
Post-BMA	258	4.9

BMA: bone-modifying agent; IV: intravenous; SC: subcutaneous; BMA timing refers to the temporal relation between invasive dental procedures (extractions and/or implant placement) and BMA therapy.

**Table 2:** Tooth extraction and MRONJ after extraction according to timing relative to BMA therapy (patient-level).

Timing group	Patients in group, n	Patients with ≥1 extraction, n (%)	MRONJ after extraction, n (%)
Pre-BMA	4,025	968 (24.1)	0 (0.0)
Intra-BMA	1,001	445 (44.5)	6 (1.35)
Post-BMA	258	35 (13.6)	2 (5.71)
Total	5,284	1,448 (27.4)	8 (0.15)*

MRONJ: medication-related osteonecrosis of the jaws; BMA: bone-modifying agent.*Eight MRONJ cases were temporally associated with extraction. Total MRONJ in the cohort was 12/5,284 (0.23%), including one case after implant placement and three spontaneous cases. Overall comparison of MRONJ after extraction among the three timing groups: χ²=27.3, degrees of freedom (df)=2, p<0.001.

**Table 3:** Dental implant placement according to timing relative to BMA therapy (patient-level).

Timing group	Patients in group, n	Patients with ≥1 implant, n (%)	Total implants placed, n
Pre-BMA	4,025	270 (6.7)	675
Intra-BMA	1,001	71 (7.1)	177
Post-BMA	258	25 (9.7)	63
Total	5,284	366 (6.9)	915

BMA: bone-modifying agent. Values for implants are counts of individual fixtures; implant outcomes are analysed at patient level.

**Table 4:** Patient-level implant failure according to timing of implant placement relative to BMA therapy (N = 366).

Timing group	Patients with implants, n	Patients with ≥1 failed implant, n (%)
Pre-BMA	270	
Intra-BMA	71	16 (5.9)
Post-BMA	25	9 (36.0)
Total	366	45 (12.3)

BMA: bone-modifying agent. Implant failure is defined at the patient level as the presence of at least one implant that became mobile, required removal because of pain/infection or lack of osseointegration, or showed progressive radiographic bone loss. Overall comparison across timing groups: χ²=39.8, df=2, p≈2.3×10-9.

**Table 5:** Multivariable logistic regression for timing of implant placement and patient-level implant failure among implant recipients (N = 366).

Predictor	Category vs reference	Adjusted OR	95% CI	p-value
Timing group	Pre-BMA vs Intra-BMA	0.16	0.08–0.33	<0.0001
	Post-BMA vs Intra-BMA	1.43	0.55–3.77	0.4645

OR: odds ratio; CI: confidence interval; BMA: bone-modifying agent.Model includes implant patients only (N=366) with timing group as the predictor and patient-level implant failure as the outcome. Intra-BMA is the reference category.

## Data Availability

The datasets used and/or analyzed during the current study are available from the corresponding author.
